# Labour market trajectories following sickness absence due to self-reported all cause morbidity—a longitudinal study

**DOI:** 10.1186/s12889-016-3017-x

**Published:** 2016-04-16

**Authors:** Pernille Pedersen, Thomas Lund, Louise Lindholdt, Ellen A. Nohr, Chris Jensen, Hans Jørgen Søgaard, Merete Labriola

**Affiliations:** Psychiatric Research Unit West, Regional Psychiatric Services West, Herning, Central Denmark Region Denmark; Institute of Clinical Medicine, University of Aarhus, Aarhus, Denmark; Public Health and Quality Improvement, Aarhus, Central Denmark Region Denmark; Institute of Clinical Research, University of Southern Denmark, Odense, Denmark; Department of Public Health and General Practice, Norwegian University of Science and Technology, Trondheim, Norway; National Centre for Occupational Rehabilitation, Rauland, Norway; Section of Clinical Social Medicine and Rehabilitation, School of Public Health, University of Aarhus, Aarhus, Denmark

**Keywords:** Return to work, Mental disorders, Sick leave, RTW-expectations, Sequence analysis

## Abstract

**Background:**

To investigate differences in return to work (RTW) and employment trajectories in individuals on sick leave for either mental health reasons or other health related reasons.

**Methods:**

This study was based on 2036 new sickness absence cases who completed a questionnaire on social characteristics, expectations for RTW and reasons for sickness absence. They were divided into two exposure groups according to their self-reported sickness absence reason: mental health reasons or other health reasons. The outcome was employment status during the following 51 weeks and was measured both as time-to-event analysis and with sequence analysis.

**Results:**

Individuals with mental health reasons for sickness absence had a higher risk of not having returned to work (RR 0.87 (0.80;0.93)). Adjusting for gender, age, education and employment did not change the estimate, however, after adding RTW expectations to the model, the excess risk was no longer present (RR 1.01 (0.95;1.08)). In relation to the sequence analysis, individuals with mental health related absence had significantly higher odds of being in the sickness absence cluster and significantly lower odds for being in the fast RTW cluster, but when adjusting for RTW expectations, the odds were somewhat attenuated and no longer significant.

**Conclusions:**

Employees on sick leave due to self-reported mental health problems spent more weeks in sickness absence and temporary benefits and had a higher risk of not having returned to work within a year compared to employees on sick leave due to other health reasons. The difference could be explained by their lower RTW expectations at baseline. This emphasises the need to develop suitable and specific interventions to facilitate RTW for this group of sickness absentees.

## Background

Sickness absence causes have different impact on the individuals’ chance of return to work (RTW) [1, 2]. Those with mental health conditions have a low RTW rate, whereas those with e.g. infectious diseases have a relatively high RTW rate [3]. Compared to other health related diagnoses, individuals on sick leave due to mental disorders have an increased number of sick leave spells and sick leave days [4, 5]. Moreover, many mental disorders are persistent and have high recurrence rates [6], are associated with increased risk of early retirement [7], and of receiving disability benefits [6] and unemployment benefits [5]. The reason for the lower RTW rate in that group may not solely be attributed to the disorder itself. It could also be explained by their lower RTW expectations [8], which are found to be a predictor for RTW [8–13].

Regardless of underlying conditions for sickness absence, the RTW process after sickness absence is complex and evolving as it covers a series of events, transitions and phases of employment status [14, 15]. The criterion of RTW is not straightforward and there are different ways of defining a RTW outcome [16–18]. Research findings can vary with the way that RTW is defined and measured. One way of defining “RTW” is to measure RTW status at a certain point in time after onset of sickness absence, for example after 3 months, 6 months or a year (i.e. a point prevalence measure). This is a convenient measure but one that may underestimate or overestimate the total effect of an employee’s work capacity, because RTW rates vary over time. Other criteria for RTW that are used in the literature include time from injury to first RTW, or the number of days lost from work after the injury. In addition to these differences, the measurement of the period until RTW may be based on actual days off work or a proxy measure such as compensation days until RTW [16, 19].

The often adapted time-to-event approach does not cover the many possible states and transitions experienced by individuals on sick leave. Employment status transitions after sickness absence have recently been studied in the Nordic countries by using multi-state models [20–23]; however sequence analysis has not previously been used to study transitions in sickness absence research. This study will evaluate the RTW measures in a Danish population on sick leave using both time-to-event analysis and sequence analysis with 51 weeks of follow-up. The aim of this study was to investigate differences in RTW and employment trajectories in individuals on sick leave for mental health reasons and individuals with other health-related reasons for sick leave.

## Methods

### Participants and design

From September 2012 to March 2014, all new cases of sickness absence exceeding 4 weeks (*n* = 4541) in the Western part of Denmark were registered. They received a questionnaire about social characteristics, RTW expectations and reasons for sickness absence. The questionnaire was originally used for an RCT study evaluating the effect of psychoeducation on RTW in individuals on sick leave [24]. No difference in relative risk of RTW during the first 6 and 12 months after inclusion was found between the intervention group and the control group [25].

The questionnaire was completed by 2788 individuals (61.4 %). Those who did not provide data on reason for sickness absence (*n* = 20), information on education (*n* = 31), employment (*n* = 123) and RTW expectations (*n* = 126) were excluded. All participants were linked to The Danish National Labour Market Authority’s DREAM database [26], which provided information about economic compensation for unemployment, sickness absence, and other kinds of social transfer income. The type of transfer payment in DREAM is recorded for each week if the person has received the benefit for one day or more. Termination of registration occurs following the first full week of not receiving any type of transfer payment. If no transfer payment is registered for a specific week, the person is considered to be self-supporting and consequently as working. In Denmark, a citizen in the workforce (employed as well as unemployed) is entitled to sickness absence compensation (at the time of this study after 4 weeks), and if the employee receives normal salary during the sick leave period, the employer receives municipal reimbursement. Data from the DREAM database is increasingly applied in research and has been validated in research in individuals on sick leave [26–28].

A total of 452 participants were not registered as being on sick leave in the DREAM database when the questionnaire was distributed and consequently they were excluded from the study. It was done to avoid misclassification and that a difference in social benefits in the study could be attributed to a difference in social benefits at baseline. Thus, the final study population consisted of 2036 individuals between 18 and 64 years of age (mean: 44.5, sd: 11.1). Data on registrations in the DREAM database was obtained from the week the questionnaire was sent and 51 weeks onwards.

### Outcome measures

The outcome variable in this paper was employment status during the 51 weeks following the questionnaire and was recorded weekly.

In the time-to-event analysis, the outcome was return-to-work, which was defined as the period (in weeks) between inclusion and the first period of 4 consecutive weeks without receiving any social benefits.

In the sequence analysis, the outcome was extended to include five different categories for labour market participation and RTW: 1) sickness absence, 2) working 3) unemployment, 4) temporary support (other than unemployment and sickness benefits), and 5) permanent support. Working was defined as the weeks with no benefits, and unemployment was defined as receiving unemployment benefits. Temporary support was defined as social benefits that are given temporarily aiming at promoting subsequent employment, e.g. public education grant, social assistance or rehabilitation benefit. Permanent support was defined as social benefits that are given on a permanent basis, where regular employment is no longer possible e.g. early retirement, public retirement pension and supported job (the Danish labour market arrangement for people with reduced ability to work and wage is partly compensated).

### Exposure variables

Self-reported reason for sickness absence was the main exposure. The participants stated in the questionnaire what they considered to be the reasons for their absence. They could report several reasons, but if they had reported anxiety, depression, other mental illness or stress and burnout, they were categorised as having “mental health reasons”, while the rest of the individuals were categorised as having “other health reasons” (e.g. musculoskeletal disorders, cancer, or chronic pain (Table 1).

**Table 1:** Reasons for sickness absence in the two exposure groups

Reasons for sickness absence	Mental health reasons *n* = 725 *n* (%)	Other health reasons *n* = 1,311 *n* (%)
Anxiety	218 (30.1)	0 (0)
Depression	405 (55.9)	0 (0)
Stress and burnout	516 (71.2)	0 (0)
Other mental illness	79 (11.0)	0 (0)
Personal problems	139 (19.2)	34 (2.6)
Psychosocial working environment	166 (22.9)	46 (3.5)
Cardiovascular or lung diseases	25 (3.5)	106 (8.1)
Infection	19 (2.6)	53 (4.0)
Chronic/diffuse pain	85 (11.7)	197 (15.0)
Cancer	16 (2.2)	66 (5.0)
Abdominal illness	32 (4.4)	63 (4.8)
Musculoskeletal disorders	74 (10.2)	799 (61.0)
Other/unclear reason	74 (10.2)	210 (16.0)

Percentages do not add up to 100 as people could report several reasons for sickness absence

### Covariates

Information about education, employment, age, gender and RTW expectations was retrieved from the questionnaire. RTW expectations were estimated by the participants as the probability of not being on sick leave after 6 months (as a percentage in whole tens from 0 to 100 %). The covariates were categorized as seen in Table 2.

**Table 2:** Baseline characteristics of the study population

Variable	Mental health reasons (*n* = 725) *n*/mean %/sd		Other health reasons (*n* = 1,311) *n*/mean %/sd		*P*-value*
Gender (female)	481	66.3	679	51.8	<0.001
Age (years)	42.3	10.6	45.7	11.2	<0.001
Highest level of education					
Primary school/Secondary school	182	25.1	405	30.9	<0.001
Tertiary education <3 years	307	42.3	617	47.1	
Tertiary education >3 years	236	32.6	289	22.0	
Employment					
Supported jobs/early age pension	24	3.3	37	2.8	<0.001
Student	43	5.9	36	2.8	
Unemployed	105	14.5	130	9.9	
Unskilled worker (e.g. cleaning)	100	13.8	263	20.1	
Skilled worker (e.g. artisan)	93	12.8	293	22.4	
White collar worker (e.g. nurse)	320	44.1	427	32.6	
Self-employed	40	5.5	125	9.5	
Recovery expectations					
0–30 %	87	12.0	90	6.9	<0.001
40–60 %	160	22.1	151	11.5	
70–90 %	180	24.8	211	16.1	
100 %	298	41.1	859	65.5	

*P-values indicate tests of differences between exposure groups by *Chi2 test* or *t*-test

### Ethical considerations

Participation was voluntary, and the study has been registered and approved by the Danish Data Protection Agency (http://www.datatilsynet.dk). The participants did not provide consent, as the data were analysed anonymously.

### Statistical analysis

Initially, a comparison of the individuals from the two exposure groups was made in relation to age, gender, education, employment and RTW expectations by means of Chi2 or t-tests.

Secondly, the pseudo value-regression approach was used to examine differences in RTW between the two exposure groups by calculating relative risk (RR) and cumulative incidence proportions (CIP) at the end of the 51 weeks of follow-up [29, 30]. CIP showed the percentages of individuals in each group who had returned to work. The allocation of the RCT study was adjusted for in all steps of the analysis [25] and thereafter, different adjustment strategies were carried out based on variables that were chosen a priori; 1) adjustment for gender and age, 2) plus education and employment and 3) plus RTW expectations. Death, emigration and receiving permanent support were considered as competing risk.

Furthermore, sequence analysis was performed, which is a statistical study of successions of states or events. A sequence is defined as an ordered list of elements (e.g. labour market status) and episodes (identical successive elements) expressed on a time axis [31, 32]. In this study, sequences showed a complete event history of labour market participation in each particular week from baseline to follow-up. The relative proportion of each of the five employment status for every week was displayed in a status proportion plot [33]. In the sequence analysis, further 18 participants were excluded due to death or emigration (four from mental health reasons and 14 from other health reasons). Thus, in those analyses, the study population consisted of 2,018 participants.

In the sequence analysis, the mean duration in weeks within a given state and the mean number of episodes of different status for the exposure groups were calculated. Differences between exposure groups were performed by using the syntax ttesti in STATA by adding the n, mean and sd for each group. This syntax was used as sequence analysis was made in long format and thus regular tests were not possible to perform.

The distributions of the sequences were compared in the two exposure groups. All individuals were divided into four groups according to their sequences; 1) only sick leave, 2) moving to continuous work, 3) having at least one episode of work, and 4) sick leave and social benefits. The different distributions of sequences were tested in a chi2 test.

A volatility indicator was defined as the proportion of work and unemployment episodes in relation to total episodes. Episodes within work and unemployment reflected a positive status of RTW or readiness to RTW. The volatility indicator indicated that the higher the value of this indicator (range 0–1), the higher the quality of the transitions [34].

An integration indicator was measured as an indicator of how quickly and to what extent the individuals re-entered employment. It was assessed as the sum of number of sequence positions where status was work, which were weighted by their position within the sequence. This indicated that the longer or more episodes in work, the higher the quality of the integration process (range 0–1) [34].

Moreover, the sequences were grouped based on optimal matching algorithms and statistical cluster analysis to find and categorize observed sequences into a smaller number of clusters [31, 35]. Optimal matching was used to measure dissimilarities between sequences by applying the Levenshtein distance measure, which measured the number of operations that were needed to transform one sequence into another [31]. Similar sequences were grouped together using hierarchical cluster analysis with Ward’s linkage [34, 35]. On the basis of these results, similar sequences were merged into eight clusters, which were named based on employment status. Afterwards, the distribution of the clusters across the exposure groups was tested by means of logistic regression. The same adjustment strategies were used as in the pseudo value analysis.

Point estimates were presented with 95 % confidence intervals. STATA/IC 11.2 (StataCorp LC, College Station, TX) was used for all statistical analyses with the SQ-ADOS to perform the sequence analyses.

## Results

A total of 725 individuals (36 %) reported mental health problems as reason for their sickness absence while 1311 (64 %) reported other health reasons. The most frequent diagnoses in the mental health group were stress and burnout, depression and anxiety, while in “other health reasons”, musculoskeletal disorders, chronic/diffuse pain and unclear reasons were most frequent (Table 1). The two exposure groups were significantly different in relation to all baseline characteristics (Table 2). Individuals with mental health as reason for the sickness absence were more often women, younger, more than 3 years of tertiary education, and had lower expectations of returning to work within the next 6 months. Furthermore, they were to greater extent white collar workers and less often unskilled or skilled workers.

A total of 56 % (95 % CI: 52;59) of those individuals who had reported mental health as reason for the sickness absence had returned to work at 51 weeks of follow-up, which was significantly lower than 67 % (95 % CI: 65;70) among those reported other health reasons (Table 3). During follow-up, individuals with mental health reasons had a significantly higher risk of not having returned to work. Even after adjusting for gender, age, education and employment, the difference was still present but somewhat attenuated. When adjusting for RTW expectations, the RR was the same in the two groups.

**Table 3:** Change of having returned to work in individuals on sick leave due to mental health or other health reasons at 1 year follow-up

Reason for sickness absence	CIP % (95 % CI)	Crude analysis ^a^ RR (95 % CI)	Adj. model 1 RR (95 % CI)	Adj. model 2 RR (95 % CI)	Adj. model 3 RR (95 % CI)
Other health reasons *n* = 1,311	67 (65;70)	1 (ref)	1 (ref)	1 (ref)	1 (ref)
Mental health reason *n* = 725	56 (52;59)	0.87 (0.80;0.93)	0.89 (0.82;0.96)	0.92 (0.85;0.99)	1.01 (0.95;1.08)

*Abbreviations*: *RR* relative risk, *CI* confidence interval, *CIP* cumulative incidence proportion, shows the percentages of individuals having returned to work

^a^Adjusted for effect of the psychoeducation intervention, Adj. model 1: Adjusted for effect of intervention, gender, and age, Adj. model 2: Adjusted as in model 1 and also for education and employment, Adj. model 3: Adjusted as in model 2 and also for RTW expectations

**Fig. 1:**
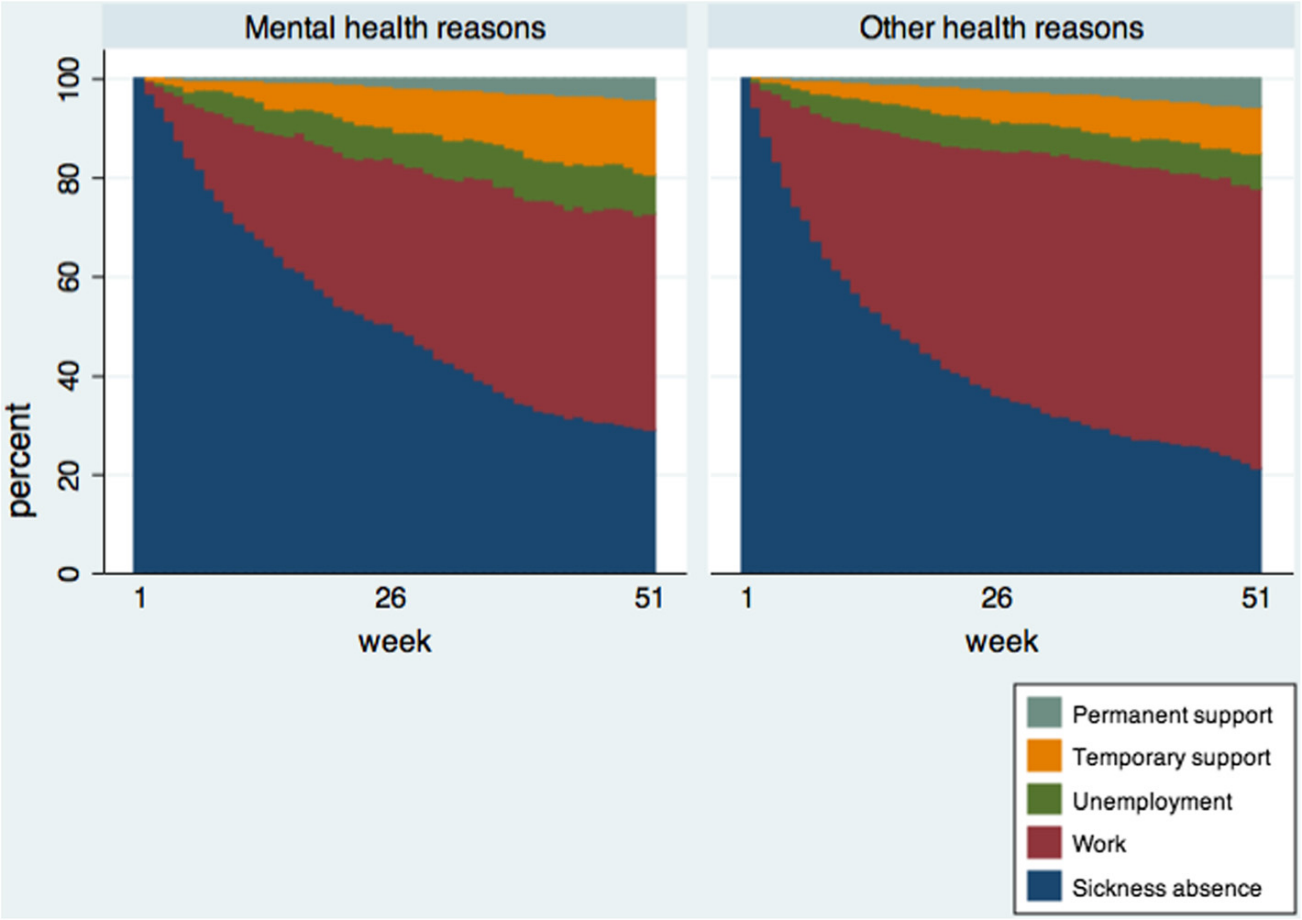
Weekly share of status by exposure groups

The status proportion plot illustrated the differences in employment status in the two exposure groups (Fig. 1). Individuals with mental health reasons had significantly more weeks of sickness absence and temporary support throughout the year compared to individuals with other health reasons (Table 4). Individuals with other health reasons had significantly more weeks of work compared to individuals with mental health reasons. No difference in the duration of unemployment and permanent support was seen between the two groups.

**Table 4:** Characteristic of labour market sequences in exposure groups

	Mental health reasons *n* = 721 Mean (sd)	Other health reasons *n* = 1,297 Mean (sd)	Diff	*p*-value*
Mean duration in				
Sickness absence	27.50 (17.57)	22.53 (17.76)	4.97	<0.0001
Work	15.96 (17.20)	22.45 (19.00)	−6.49	<0.0001
Unemployment	3.38 (6.69)	2.80 (7.12)	0.58	0.07
Temporary support	4.18 (9.49)	2.91 (8.14)	1.27	<0.01
Permanent support	0.98 (5.42)	1.31 (6.23)	−0.33	0.24
Mean number of episodes in				
Sickness absence	1.32 (0.78)	1.37 (0.99)	−0.05	0.25
Work	1.02 (1.07)	1.17 (1.21)	−0.15	<0.01
Unemployment	0.62 (1.14)	0.50 (1.12)	0.11	0.03
Temporary support	0.52 (0.97)	0.38 (0.86)	0.14	<0.001
Permanent support	0.05 (0.22)	0.06 (0.25)	−0.02	0.17
Mean number of episodes (total)	3.52 (2.90)	3.48 (2.93)	0.04	0.76
Mean number of different elements in sequence	2.32 (0.97)	2.27 (0.84)	0.05	0.20
Volatility indicator	0.37 (0.24)	0.40 (0.22)	−0.04	<0.001
Integration indicator	0.36 (0.38)	0.49 (0.40)	−0.13	<0.0001

*Abbreviations*: *SD* standard deviation

*p-values generated by means of the “ttesti” syntax in STATA

The group with other health reasons had significantly more episodes of work, whereas individuals with mental health reasons had more episodes of unemployment and temporary support. No differences in the mean number of episodes in the five employment status or the mean number of different elements in the sequences were seen in the two exposure groups (Table 4). The range of episodes in the follow-up period was 1–23 in the group of mental health reasons and 1–26 in the group of other health reasons.

Individuals with mental health reasons had a significantly lower volatility indicator and integration indicator compared to individuals with other health reasons.

During the follow-up period, there were a total of 181 different sequences in the group with mental health reasons and 238 in the group with other health reasons. The most frequent sequence in both groups was going from sickness absence to continuous work, as it happened to 195 individuals (27.0 %) from the group of mental health reason and 509 individuals (39.2 %) from the group of other health reasons. The second most frequent sequence in both groups was staying in sickness absence throughout the study period. Thus, a total of 144 individuals (20.0 %) with mental health reasons and 174 individuals (13.4 %) with other health reasons were on sick leave for 51 weeks. In relation to the rest of the participants, a total of 279 individuals (38.7 %) with mental health reasons had at least one episode of work compared to the 477 individuals (36.8 %) with other health reasons. Moreover, 103 (14.3 %) and 137 (10.6 %) were on sick leave and social support in the group of mental health reasons and in the group of other health reasons, respectively. A chi2 test showed a significant difference between the exposure groups in the distribution of the sequences (*p* <0.0001).

**Fig. 2:**
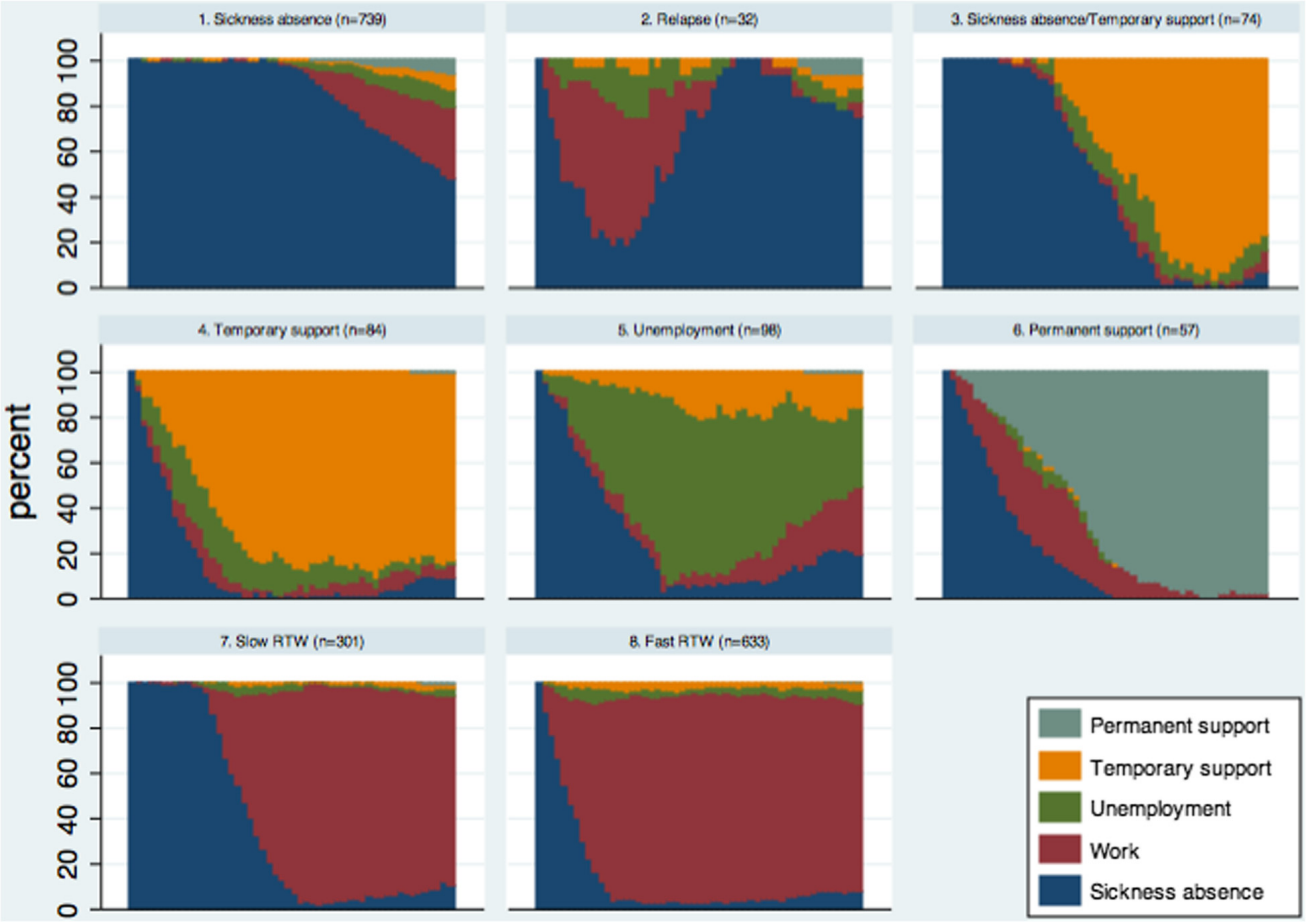
Aggregated shares of employment status by clusters

The eight clusters, which were merged on the basis of similar sequences, displayed aggregated shares of employment status (Fig. 2). Three of the clusters (5, 7 and 8) displayed work-oriented trajectories while two clusters (1 and 2) indicated continuous sickness absence or relapse into sickness absence. Only one cluster (6) showed a permanent withdrawal from the labour market while two clusters (3 and 4) displayed general or partial temporary support.

Individuals with mental health reasons had significantly higher odds for being in the sickness absence cluster and significantly lower odds for being in the fast RTW cluster after adjusting for gender, age, education and employment; however, when adjusting for RTW expectations, the odds were somewhat attenuated and no longer significant (Table 5). Moreover, the individuals with mental health reasons had significantly higher odds for being in the relapse cluster, although the number of observations was rather small. Also, the individuals with mental health reasons had marginally lower odds for being in the slow RTW cluster; however, after adjusting for RTW expectations the OR was close to 1.

**Table 5:** Sickness absence reason and risk of being in eight different clusters

Clusters	Mental health reason *n* = 721 *n* (%)	Other health reason *n* = 1,297 *n* (%)	Crude analysis^a^ OR (95 % CI)	Adj. model 1 OR (95 % CI)	Adj. model 2 OR (95 % CI)	Adj. model 3 OR (95 % CI)
1. Sickness absence						
	317 (44.0)	422 (32.5)	1.35 (1.10;1.65)	1.30 (1.06;1.59)	1.31 (1.06;1.60)	1.05 (0.85;1.31)
2. Relapse						
	17 (2.4)	15 (1.2)	2.69 (1.31;5.52)	2.69 (1.29;5.59)	2.65 (1.27;5.52)	2.77 (1.31;5.87)
3. Sickness absence/temporary support						
	39 (5.4)	35 (2.7)	1.92 (1.17;3.16)	1.60 (0.97;2.65)	1.55 (0.94;2.56)	1.32 (0.80;2.18)
4. Temporary support						
	37 (5.1)	47 (3.6)	1.45 (0.91;2.32)	1.12 (0.70;1.82)	1.11 (0.68;1.79)	1.12 (0.69;1.82)
5. Unemployment						
	32 (4.4)	66 (5.1)	0.83 (0.52;1.32)	0.80 (0.50;1.27)	0.77 (0.48;1.23)	0.79 (0.49;1.27)
6. Permanent support						
	17 (2.4)	40 (3.1)	0.91 (0.50;1.66)	1.74 (0.90;3.39)	1.61 (0.83;3.12)	1.43 (0.73;2.80)
7. Slow RTW						
	104 (14.4)	197 (15.2)	0.87 (0.66;1.14)	0.93 (0.70;1.24)	0.92 (0.69;1.23)	1.03 (0.77;1.38)
8. Fast RTW						
	158 (21.9)	475 (36.6)	0.63 (0.50;0.78)	0.66 (0.52;0.82)	0.67 (0.53;0.84)	0.84 (0.66;1.07)

Reference group: Other health reasons

*Abbreviations*: *OR* odds ratio, *CI* confidence interval

^a^Adjusted for effect of the psychoeducation intervention, Adj. model 1: Adjusted for effect of intervention, gender and age, Adj. model 2: Adjusted as in model 1 and also for education and employment, Adj. model 3: Adjusted as in model 2 and also for and RTW expectations

## Discussion

### Main results

Individuals on sick leave due to mental health reasons spent more weeks on sickness absence and in temporary support and less weeks on work compared to individuals with other health reasons for sick leave. Moreover, fewer of the individuals on sick leave due to mental health reasons had returned to work during the 51 weeks of follow-up, compared to the individuals with other health reasons.

Also the chance of having returned to work was lower for individuals with mental health reasons when adjusting for gender, age, education and employment status but after adjusting for RTW expectations, the chance was the same in the two groups. Moreover, individuals with mental health reasons had higher odds of being in the “sickness absence” cluster and a lower odds of being in the “fast RTW” cluster, but the difference was attenuated after adjusting for RTW expectations.

### RTW expectations

The results show that RTW expectations can be considered a confounder in the effect of health reasons for RTW. Individuals with mental health reasons returned to work later than individuals with other health reasons, but after adjusting for RTW expectations both exposure groups were found to return to work at the same time. Other studies have also found RTW expectations to be a predictor of RTW in both individuals on sick leave due to mental and physical disorders i.e. a positive RTW expectation predict a shorter time to RTW [8–13]. It has been speculated that positive RTW expectations represent the self-efficacy of the employee, i.e. the belief an individual has in his/her own capacity to perform a specific behaviour successfully, in this case in relation to RTW [8, 10]. Furthermore, bad mental health and low RTW expectations could be influenced by the same problems, i.e. problems meeting demands at work or at home, social problems at work or other work-related factors may have triggered both mental health problems and low RTW-expectations if the prospects of solving these problems seem low.

Individuals with other health reasons had a higher level of RTW expectations than individuals with mental health reasons. This has also been confirmed in a study by Huijs et al. [8]. Another possible explanation could be that the stigmatization of mental health problems in the workplace is high, and therefore the employees might avoid their workplace and receive less support from their colleagues and supervisor, making it seem less likely to return to work. A third explanation of the lower RTW expectations among individuals with mental health reasons could be influenced by their psychological symptoms like hopelessness, discourage and reduced self-confidence. These symptoms likely reduce the belief of RTW.

### Transitions in the RTW process

The maximum number of episodes for one individual was 23 in the group of mental health reasons and 26 in the group of other health reasons. This shows that the RTW process for individuals on sickness absence benefits may be long and complex [15, 36], which is in line with previous Nordic studies using multi-state models [20–23]. It also emphasises the need to analyse RTW as a process [14, 15], and not only at a single point in time [37, 38]. The advantage of this approach is that it provides a more complete picture of RTW and employment trajectories and therefore, a more complete understanding of the impact of disability on the employee’s life and well-being [36, 39].

During the last 10 years, transitions of states have been used in the research of sickness absence by means of multi-state models [20–23]. Pedersen et al. showed the transitions for Danish individuals on sick leave and with 4 year follow-up [22]. They included the states; work, unemployment, sickness absence, and disability pension, and identified predictors for each of the different transitions. Three Norwegian studies have used multi-state models to analyse the transitions of states [20, 21, 23]. Lie et al. applied three different states that low back pain patients could be in after an intervention; recovery (RTW), sick leave benefits, or disability pension [20], while Gran et al. also included partial sick leave and work assessment allowance [23]. Oyeflaten et al. extended the model to include eight different categories for social benefits or return to work over a 4 year period [21]. Only Oyeflaten et al. included categories on varying types of social benefits, whereas Pedersen et al., Lie et al. and Gran et al. mostly looked at disability benefits besides work and sickness absence. To be able to show a more realistic picture of the transitions, it is relevant to include all types of social benefits.

### Strength and limitations

The prospective design of the study and the record linkage of the cohort data with sickness absence data from DREAM added to the strengths of this study. The study had complete follow-up of weekly employment status due to full coverage of registers of social benefits and the information is considered valid [26]. Moreover, this study included sequence analysis to look at transitions besides the more traditional time-to-event outcome. Using the method has given an overview of the life course after the start of the sickness absence period. Sequence analysis is considered an exploratory method rather than a method for hypothesis testing, which means that sequence analysis cannot answer the question of causality. Due to this, sequence analysis is best used in combination with other methods, and cannot replace methods like event history models [40].

There is no clear agreement about how long a follow-up period is needed to get the best measurement of the effect on work and benefits after sick leave [16, 41]. Previous studies using process analyses have used a longer follow-up period, i.e. 3–4 years [20–23]. In this study, only 51 weeks of follow-up was applied which reduces the complexity of the sequences as e.g. 20 % with mental health reasons and 13 % with other health reasons were still on sick leave and thus, had not changed states. Thus, a longer follow-up period would have been preferable, as Oyeflaten et al. concluded that several years are needed to get an adequate picture of the RTW outcome [21].

The frequency of mental disorders in RTW research has been found to be underestimated [4, 42, 43]. Therefore, the grouping of exposure may cause misclassification if the individuals are not true about reporting the sickness absence reason. However, as the questionnaire was sent in relation to an RCT study for individuals with mental health problems, it is considered a minor issue. Moreover, misclassification in relation to the outcome may occur as a new sickness absence period is registered only if it is longer than 4 weeks. Thus, the short term sickness absence periods may be underestimated, which at the same time may overestimate the participation in work. Also, DREAM provides no information on whether an individual is actually working or not. When data from DREAM are used in research studies, work is categorised as those weeks which the individual does not receive any benefits. However, DREAM data have been validated in the context of sick leave [26, 28].

Some studies have divided the sickness absence reasons into mental, physical and co-morbidity and found that co-morbidity was associated with longer time until RTW than only reporting physical or mental problems [8, 44]. In this study, individuals with co-morbidity were not categorized separately, as it was not the aim of the study. Moreover, only co-morbidity that was due to the sickness absence was reported. Therefore, the degree of co-morbidity in this study is unknown.

Another limitation derives from the relatively low response rate (61.4 %). The relationship between sickness absence reasons and employment status may have been different in non-responders, and thus could change the estimates. If individuals on sick leave due to mental health reasons to a greater extent did not response to the questionnaire and at the same time were on sick leave for a longer time period, we may have underestimated the association between sickness absence for mental health reasons and RTW. Moreover, some of the clusters in Fig. 2 included few observations which will give rise to a large random variation.

### Generalization

Comparison between studies may be difficult due to the large variation between countries in the regulation of sick leave compensation and social benefits. Within the Nordic countries, the social security systems are relatively similar and make comparisons feasible [45]. Our findings may, therefore, be generalized to the Nordic countries. However, we see no reason why the longer sickness absence periods and lower RTW expectations for those with mental health problems than for those with other health problems should not be similar in other Western countries.

## Conclusion

Employees on sick leave due to self-reported mental health problems spent more weeks in sickness absence and temporary benefits and had a higher risk of not having returned to work within a year compared to employees on sick leave due to other health reasons. The difference could be explained by their lower RTW expectations at baseline. This emphasises the need to develop suitable and specific interventions to facilitate RTW for this group of sickness absentees.

## Availability of data and materials

The dataset supporting the conclusions of this article can not be shared as it contains information on social transfer income and sickness absence diagnoses.
